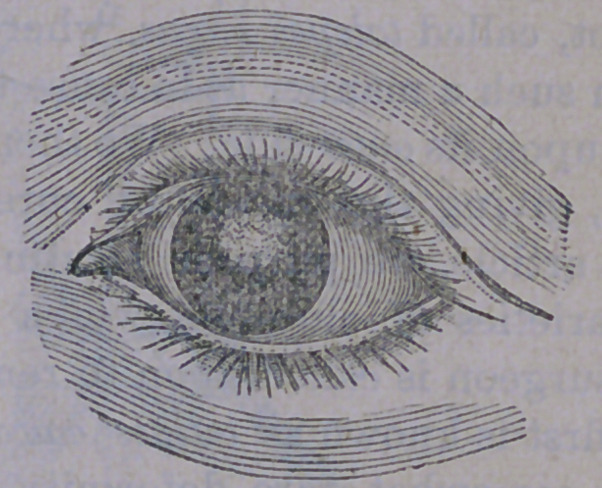# The Cornea

**Published:** 1875-01

**Authors:** 


### THE CORNEA.

The *cornea* is the clear, transparent, and prominent portion of the eye, immediately in front, and has been somewhat aptly compared to the crystal in a watch. By reason of its exposed position, it is subjected to accidents and injuries. Cinders from the locomotive, when traveling, are apt to find lodgement upon its surface, often becoming so firmly imbedded as to render their dislodgement extremely difficult. Artizans—blacksmiths, machinists, and others, frequently have particles of steel or iron fly into their eyes and adhere to the cornea, often producing very serious wounds. Children sometimes thrust forks, scissors or pen-knives in their eyes, or injure them by exploding percussion caps on old pistols, or by means of a hammer. Such injuries are often fatal to vision, exciting such inflammation in the substance of the cornea as to completely destroy its clearness. Often, more injury is done the cornea by the bunglesome attempts at removal of cinders, particles of steel, &c., by inexperienced hands, than would have resulted from the continued presence of such particles.

Inflammation of the cornea may result from any of the causes enumerated, or through scrofulous or other constitutional diseases. Its presence is characterized by sensitiveness to light, extreme lachrymation, and a haziness of its surface.

The vessels are engorged with blood, become very numerous and completely encircle the cornea, like a net-work, as is seen in the engraving. Soon, the haziness becomes more defined, until complete opacity occurs, entirely excluding the light. We are thus careful to note the progress of the disease, because its consequences are so little understood by the public, and many eyes are lost, that might have been saved, had the symptoms enumerated been met in season, by careful treatment, at the hands of competent ophthalmic surgeons.

Inflammation of the cornea is apt to arise in children, suffering from the eruptive fevers—measles, scarlatina, and small-pox. In adults, it often accompanies rheumatism, and syphilitic taint. If the inflammation is allowed to run its course, it will terminate either in complete opacity and destruction of the cornea, in which case blindness is the result, or, ulceration may occur, which, when healed, will leave an opaque spot corresponding to the size of the ulcer. An illustration of opacity from an ulcer upon the cornea, is here given :

showing a white spot just over the pupil, greatly interfering with vision. Such an eye, of course, must be nearly blind. The only remedy for an eye so obstructed, is to undergo an operation for *artificial pupil*, (fully described in the April No., 1871) by means of which the vision can be almost wholly restored.

Let me again impress upon our readers the necessity for prompt treatment in all inflammations of the cornea, if they would avert the sad consequences here portrayed. Above all things, avoid patent eye-waters. All such nostrums contain more or less preparations of lead, ("sugar of lead" usually), which is fatal to an ulcer upon the cornea. The great majority of opacities of the cornea with which we come in contact, have been produced by these vile nostrums.

We are always loath to recommend any treatment for diseases of the eye, realizing how very difficult or impossible it is to indicate a course of medication that would be applicable in all cases. In case no ophthalmic surgeon is near enough to be consulted, a safe plan would be to use poppy fomentations to the eyes, giving gentle laxatives occasionally, and keeping the patient in a perfectly dark room. Leeching, cupping and blistering is dangerous, generally aggravating the complaint. If the disease depends upon a scrofulous taint, quinine and iron, with generous diet should be given. If from syphilitic taint, iodide of potassium and bichloride of mercury are indicated, in addition to the local treatment advised. Of course, these medicines can only be given under the advice of the family physician, in such doses as his judgment suggests. We only throw out these hints to assist the general practitioner whose experience has necessarily been limited in the management of such cases.

## Figures and Tables

**Figure f1:**
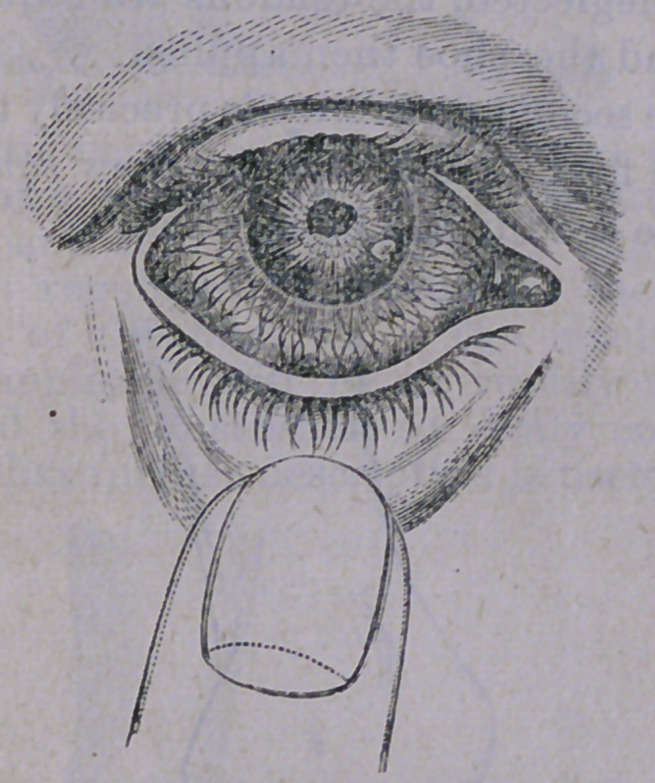


**Figure f2:**